# Antibody-drug conjugates for precision oncology in cholangiocarcinoma

**DOI:** 10.17179/excli2025-8952

**Published:** 2025-11-14

**Authors:** Parteek Prasher, Mousmee Sharma

**Affiliations:** 1 Department of Chemistry, University of Petroleum & Energy Studies (UPES), Dehradun, 248007, India; 2 Department of Chemistry, Uttaranchal University, Dehradun, 248007, India

## ⁯

Cholangiocarcinoma (CCA), a rare and aggressive malignancy of the bile ducts, presents significant clinical and healthcare challenge due to its asymptomatic progression, diagnostic complexity, and limited therapeutic options. In recent years, a deeper understanding of its molecular heterogeneity has revolutionized the management approach through precision medicine. Actionable genetic alterations such as Fibroblast Growth Factor Receptor 2 (FGFR2) fusions, Isocitrate Dehydrogenase 1 (IDH1) mutations, serine/threonine-protein kinase B-Raf. (BRAF) mutations, and human epidermal growth factor receptor 2 (HER2) overexpression have led to the development and Food and Drug Administration (FDA) approval of several targeted therapies including pemigatinib, ivosidenib, futibatinib, durvalumab, and zanidatamab. While these advances mark a significant step forward, they are only applicable to a minority of patients and are further restricted by mechanisms of primary and acquired resistance, limited duration of response, and adverse events. Antibody-drug conjugates (ADCs) offer a transformative opportunity by uniting selective antibody targeting with the cytotoxic power of small-molecule payloads. This strategy enables precision delivery within the tumor microenvironment, exploiting mechanisms such as protease-, pH-, or redox-sensitive linkers to achieve controlled drug release (Riccardi et al., 2023[4]). Recent preclinical advances highlight ADCs directed against Glypican-1 (GPC1), CD44 variant 5 (CD44v5), Intercellular Adhesion Molecule 1 (ICAM1), HER2, and Trop-2, showing potent antitumor activity, immune modulation, and even synergism when paired with checkpoint inhibitors. These findings collectively position ADCs as versatile agents capable of addressing tumor heterogeneity, overcoming therapeutic resistance, and broadening the scope of precision oncology in CCA. While clinical validation is still in its infancy, the momentum of translational research underscores ADCs as a next-generation therapeutic class poised to reshape the management of this devastating cancer. This article provides an overview of the current progress, challenges, and future directions of ADC-based strategies in cholangiocarcinoma.

ADCs represent a precision medicine approach for the management of CCA where a cytotoxic payload/ drug is covalently attached to tumor cell specific surface antigens via labile bonds which degrade under tumor microenvironment (Kuwatani and Sakamoto, 2023[3]). The cleavable bonds include enzyme-responsive peptide linkers which are cleaved in lysosomes (cathepsin B), acid-responsive hydrazone linkage, which is cleaved in lysosomes and endosomes, reducible linkers such as disulfide which is cleaved under high intracellular glutathione in cytosol of cancer cells, and specialized enzyme-triggered linkers such as β-glucuronide, phosphatase-sensitive, esterase-sensitive carbamate/ester linkages. Apart from this, non-cleavable linkers also integrate tumor specific antibodies with the cytotoxic drug, which on their internalization to the cells are transported to lysosomes, where the proteases degrade the antibody to release the payload drug still attached to the linker and single amino acid residue derived from the antibody. In this case, the cytotoxic drug exerts its effect in cytosol of target cancer cells. While there are no clinically approved ADCs so far for specifically targeting CCA, the preclinical studies provide rationale for translating ADCs for targeting GPC1, CD44v5, and HER2 into early phase clinical trials.

### Glypican-1 (GPC1)-targeted ADCs

GPC1 is a heparan sulphate proteoglycan attached to cell-surface via GPI anchor which is implicated in tumor signaling and angiogenesis. Immunohistochemical analysis on 49 extrahepatic CCA specimens by Yokota et al. (2021[7]) reported that 47 % of these (23 patients) showed high expression of GPC1 which correlates with worse disease-free survival (DFS) and overall survival (OS) with p < 0.05, 49 % (24 patients) showed low expression of GPC1, and only 4 % (2 patients) were found to be GPC1 negative. Notably, GPC1 was also found to be expressed in tumor-associated blood vessels in CCA and not in the normal vasculature of healthy tissues, thereby indicating its role as oncologic biomarker (Yokota et al., 2021[7]).

Anti-GPC1 monoclonal antibody with high internalization efficiency was conjugated to monomethyl auristatin F (MMAF), a highly potent microtubule-inhibiting drug, via maleimidocaproyl-valine-citrulline-PABC (mc-vc-PABC) peptide linker. The cathepsin B-cleavable linker ensures stability of ADC in systemic circulation and a targeted release of the payload drug in lysosomes. The reported GPC1-ADCs showed strong *in vitro* cytotoxicity against GPC1-positive CCA cell lines KKU-055 and KKU-100. Similarly, *in vivo* experiments on GPC1-positive xenograft mouse models showed that GPC1-ADCs achieved a significant, dose dependent inhibition of tumor growth as compared to the control ADC. The GPC1-knockout tumor xenografts showed a reduction in the direct antitumor activity, however GPC1 was still present on tumor-associated endothelial cells, on which the reported ADCs induced cell cycle arrest in G2/M phase thereby indicating a secondary anti-angiogenic effect. Overall, the above preclinical data showed that targeting GPC1 by ADC serves as a potential therapy for GPC1-positive CCA (Yokota et al., 2019[6], 2021[8]).

### CD44 variant 5 (CD44v5)-targeted ADCs

Intrahepatic cholangiocarcinoma (ICC), the second most frequently occurring primary hepatic malignancy is characterized by limited therapeutic interventions with poor patient outcomes. It has been reported that the variant isoforms of cell surface glycoprotein CD44 are selectively expressed in ICC cells, hence making it a desirable target of ADC-based therapy. Specifically, CD44 variant 5 (CD44v5) has been observed to be expressed on the surface of most ICC tumors. Apparently, Bei et al. (2023[1]) developed CD44v5-targeted ADC, namely H1D8-drug conjugate (H1D8-DC) which comprises of humanized anti-CD44v5 monoclonal Antibody conjugated to a known microtubule inhibitor monomethyl auristatin E (MMAE) via cleavable valine-citrulline based linker, which is lysed by lysosomal cysteine proteases. The reported drug conjugate demonstrated an efficient binding to the antigen and internalization to CD44v5-expressing cells. Owing to a high expression of cathepsin B in ICC cells, the valine-citrulline based linker in drug conjugate is cleaved to preferentially release the payload in cancer cells which results in cytotoxic effects at picomolar concentration *in vitro *with negligible impact on normal healthy cells. *In vivo* studies on animal models with patient-derived ICC tumors suggested a pronounced regression in tumor without any visible signs of toxicity thereby suggesting CD44v5 as an anticipated target in ICC (Bei et al., 2023[1]).

### ICAM1-targeted ADCs

ICAM1 is a transmembrane glycoprotein of immunoglobulin superfamily serves as an adhesion molecule and signal receptor and is associated with the regulation of the survival and spreading of tumor cells. Serum ICAM1 has been reported as a biomarker for an early diagnosis of CCA however its therapeutic potential in the disease is not fully explored. Zhu et al. (2023[9]) developed ICAM1 ADCs consisting of ICAM1 antibodies with DX-8951 derivative (DXd) and MMAE via lysosomal cathepsin proteases-cleaved valine-citrulline (Val-Cit) dipeptide linker, and Gly-Gly-Phe-Gly (GGFG) tetrapeptide linker. The immunohistochemical (IHC) staining experiments were performed in 78 human CCA tumor tissues from both iCCA (42.5 %) and eCCA (29 %) patients. Statistical analysis indicated that CCA patients showed 37 % higher expression of ICAM1^+^ as compared to the normal bile duct tissues and liver tissues in the corresponding para-cancerous tissues. Fluorescence imaging results showed that ICAM1 antibodies were initially bound to the ICAM1 antigens on the surface of HuCCT1 (iCCA) and SK-ChA-1 (eCCA) cells, which were eventually internalized by both the CCA cells as quantified via flow cytometry assays. Results showed that ICAM1 antibodies showed 60 % internalization efficiency in HuCCT1 cells, while more than 40 % of the antibodies were endocytosed in SK-ChA-1 and QBC939 cells with HCCC-9810 cells having a lower rate (24 %) of endocytosis. These mediation of antibody endocytosis by ICAM1 protein present on CCA cells surface highlights its importance as a potential target of ADCs for CCA management (Zhu et al., 2023[9]).

### HER2-targeted ADCs and combination strategies

Recently, a preclinical investigation by Wang (2025[5]) demonstrated the antibody drug conjugate of trastuzumab which binds to HER2 protein on tumor cells, with deruxtecan which is a potent topoisomerase 1 inhibitor payload, via a cleavable tetrapeptide-based linker. The reported ADC showed synergistic inhibition of CCA cells in combination with monoclonal antibody durvalumab which serves as an immune checkpoint inhibitor. Dose-dependent cell proliferation assays on FRH-0201 and HuCCT-T1 cells and patient derived cell models showed that the combination of these agents showed a superior anti-proliferative activity as compared to their individual potential. The flow cytometry experiments further confirmed apoptosis owing to an upregulation of pro-apoptotic markers including Caspase-3, Bax and reduced Bcl-2 while cell cycle analysis showed elevated levels of p21 and decreased cyclin-dependent Kinase 2** (**CDK2), and c-Myc. Mechanistically, the treatment with these agents led to a suppression of p38 Mitogen-Activated Protein Kinase (MAPK) pathway, which apparently reduces the expression of Early Growth Response 1 (EGR1) at both mRNA and protein levels. *In vivo* studies on human CCA xenograft bearing mice experienced a prolonged survival on treatment with the above combination of ADC and monoclonal antibody, which suggested that a dual blockade of PD-L1 and HER2 targets by durvalumab and T-DXd, respectively, controls p38 MAPK-mediated downregulation of EGR1 which triggers tumor cell death (Wang, 2025[5]).

### Trop-2-targeted ADCs

In another recent report, Hosni et al. (2025[2]) developed Trop-2 targeting ADC, Sacituzumab Govitecan which delivers payload directly to Trop-2 expressing tumor cells via cleavable CL2A/ carbonate linker, which is cleaved under acidic conditions found in tumor microenvironment. The overexpression of Trop-2 in cholangiocarcinoma correlates with poor prognosis, which makes it a desirable target in CCA management. An ongoing Phase II SIGNA trial I is investigating the CCA patients who received the reported ADC after at least one prior systemic therapy with objective response rate as the primary endpoint and secondary measures including progression-free survival, disease control, and safety, and its outcomes may offer a novel, targeted therapeutic intervention in CCA (Hosni et al., 2025[2]).

### Conclusion

ADCs have emerged as a rational and highly adaptable therapeutic modality for CCA, addressing the unmet need created by late-stage diagnoses, poor response rates to conventional therapies, and tumor heterogeneity. The preclinical validation of targets such as GPC1, CD44v5, ICAM1, HER2, and Trop-2 demonstrates that ADCs can exploit tumor-specific antigens and microenvironment-responsive linkers to deliver potent cytotoxins with enhanced precision and reduced systemic toxicity. Innovations in linker chemistry, payload diversification, and combination strategies with immunotherapies further expand their therapeutic potential, offering avenues to overcome resistance mechanisms and augment immune-mediated tumor clearance. Nonetheless, challenges including antigen heterogeneity, limited penetration within the desmoplastic stroma, optimization of drug-to-antibody ratios, and the development of robust companion diagnostics remain critical barriers to translation. Collectively, the convergence of advanced molecular targeting, rational design of conjugates, and integrative treatment strategies underscores ADCs as a transformative platform poised to reshape precision oncology for CCA, pending rigorous clinical validation.

## Notes

Parteek Prasher and Mousmee Sharma contributed equally as first author.

## Declaration

### Conflict of interest

The authors declare no conflict of interest.

### Artificial Intelligence (AI) - Assisted Technology

The authors declare that they have not used artificial intelligence for the writing of the manuscript.
